# Determinants of Change in Physical Activity in Children 0–6 years of Age: A Systematic Review of Quantitative Literature

**DOI:** 10.1007/s40279-016-0656-0

**Published:** 2016-12-17

**Authors:** Kathryn R. Hesketh, Claire O’Malley, Veena Mazarello Paes, Helen Moore, Carolyn Summerbell, Ken K. Ong, Rajalakshmi Lakshman, Esther M. F. van Sluijs

**Affiliations:** 1 MRC Epidemiology Unit and Centre for Diet and Activity Research, University of Cambridge, Cambridge, UK; 2 UCL Institute of Child Health, 30 Guildford Street, London, WC1N1EH UK; 3 School for Medicine, Pharmacy and Health, Durham University, Durham, UK; 4 Fuse UKCRC Centre for Translational Research in Public Health, Durham, UK; 5 Cambridge Institute of Public Health, School of Clinical Medicine, University of Cambridge, Cambridge, UK

## Abstract

**Background:**

Understanding the determinants of children’s health behaviours is important to develop successful behaviour-change interventions.

**Objective:**

We aimed to synthesise the evidence around determinants (‘preceding predictors’) of change in physical activity (PA) in young children (0–6 years of age).

**Methods:**

As part of a suite of reviews, prospective quantitative studies investigating change in physical activity in children aged 0–6 years were identified from eight databases (to October 2015): MEDLINE, Embase, CINAHL, PsycINFO, Web of Knowledge, British Nursing Index, Applied Social Sciences Index and Abstracts, and Sociological Abstracts. Determinants and direction of association were extracted, described and synthesised according to the socio-ecological model (individual, interpersonal, organisational, community, policy).

**Results:**

Forty-four determinants, predominantly in the interpersonal and organisational domains, were reported across 44 papers (six prospective cohort, 38 interventional); 14 determinants were assessed in four or more papers. Parental monitoring showed a consistent positive association with change in PA; provider training was positively associated with change in children’s moderate-to-vigorous PA only. Five (sex, parental goal setting, social support, motor skill training and increased time for PA) showed no clear association. A further seven (child knowledge, parental knowledge, parental motivation, parenting skills, parental self-efficacy, curriculum materials and portable equipment) were consistently not associated with change in children’s PA. Maternal role-modelling was positively associated with change in PA in all three studies in which it was examined.

**Conclusions:**

A range of studied determinants of change in young children’s PA were identified, but only parental monitoring was found to be consistently positively associated. More evidence dealing with community and policy domains from low-/middle-income countries and about lesser-explored modifiable family- and childcare-related determinants is required.

**International Prospective Register for Systematic Reviews (PROSPERO) Registration Number:**

CRD42012002881.

**Electronic supplementary material:**

The online version of this article (doi:10.1007/s40279-016-0656-0) contains supplementary material, which is available to authorized users.

## Key Points

**Table:** 

Forty-four determinants of change in young children’s physical activity were assessed across 44 papers, predominately in the intrapersonal, interpersonal and organisational domain.
Although 14 determinants were assessed in four or more studies, only parental monitoring was consistently positively associated with change in physical activity and provider training associated with change in moderate-to-vigorous physical activity.
Evidence in community and policy domains, and from low-/middle-income countries, is required.

## Background

By the age of 5 years, over one in five children are overweight or obese the UK and USA [1, 2]. Obesity in childhood is associated with a range of unfavourable outcomes including type 2 diabetes, hyperlipidaemia and psychosocial problems [3], with obesity known to track and be associated with unfavourable outcomes in adulthood [4, 5]. Early childhood is a period of rapid growth and development, and the preschool years (defined here as up to the age of 6 years) are therefore ideal to both prevent and reverse unhealthy weight gain, by establishing healthy habits and behaviours.

As a result, interventions aiming to effect positive dietary, physical activity and sedentary behaviour change have been developed to prevent or halt obesity in the preschool years [6–9]. However, with a few notable exceptions [10–12], many of these interventional studies showed small effects which are not sustained over time, or have no effect at all [6–9]. One difficulty in establishing the reasons for a lack of intervention success is that multiple behaviours are often targeted simultaneously [8, 9]. However, as each health behaviour has an independent significant impact on children’s health [13, 14], it is important to establish the most important determinants of each individual behaviour, and therefore how they may differ across behaviours. The socio-ecological model (SEM) [15] is a commonly used framework for categorising levels of influence on behaviours [16, 17], classifying them into five broad categories: individual; interpersonal, organizational, community and public policy. By grouping potential influences on behaviour in this way, commonalities and differences can be identified and subsequently used to develop more targeted interventions to effectively change children’s health behaviours [18].

In addition to consuming a balanced nutritious diet, children up to the age of 5 years are recommended to engage in 180 min of physical activity daily [19, 20]. Higher levels of physical activity are associated not only with decreased adiposity in preschool-aged children but also positively associated with motor skill development, psychosocial health and decreased cardio-metabolic risk prospectively [13]. Cross-sectional studies in older preschool-aged children (2 years and over) also indicate that increased physical activity is linked to better gross motor control [21] and improved social skills [22]. Yet despite the importance of physical activity for young children’s health and development [13], studies suggest that young children do not engage in sufficient levels of physical activity [23].

In order to specifically increase physical activity in targeted interventions, it is important to establish which factors influence activity behaviour [24]. A number of systematic reviews have been conducted to examine the associations between cross-sectional factors (‘correlates’) and young children’s physical activity [16, 25, 26]. A broad range of correlates have been investigated, including demographic, biological, environmental, social and psychological influences. Although conclusions about the influences on physical activity differ between reviews [25, 27], there is a suggestion that familial influences [16, 25, 26], time spent outside [25] and elements in the physical environment [25, 27] may be associated with increased activity in preschoolers. An additional review [28] that included cross-sectional studies and a small number prospective cohorts also suggests that home influences may be key for young children’s physical activity. However, it is difficult to draw firm conclusions about causality from cross-sectional studies. It is therefore necessary to use evidence from both prospective and interventional studies as these provide the best evidence to establish the longitudinal predictors (or ‘determinants’) of change in young children’s physical activity, and to aid understanding of how to effect positive behaviour change.

This systematic review is part of a suite of reviews exploring the determinants of obesogenic behaviours in children aged 0–6 years (focussed on fruit and vegetable intake, sugar-sweetened beverages and unhealthy diet intake, physical activity and sedentary behaviour) [29, 30]. It aims to synthesise the quantitative literature from prospective and interventional studies to ascertain the determinants (a ‘preceding predictor’) of change in physical activity in young children. It also aims to establish which (modifiable) determinants are associated with change; at which levels of influence these factors operate (i.e. individual, family, childcare setting, community or policy level); and where gaps in the literature exist for future research.

## Methods

The protocol for this review project has been described previously [29]. The International Prospective Register for Systematic Reviews (PROSPERO) Registration number is CRD42012002881. Following established criteria for the rigorous conduct and reporting of systematic reviews [31, 32], this review was carried out in three stages [33, 34]. One search (led by HM) was conducted to identify studies across all reviews; at the data-extraction stage, smaller teams led each of the reviews focusing on specific behaviours of interest [i.e. physical activity (review lead: KH), fruit and vegetable consumption (COM), and sugar-sweetened beverages (VP)]. KH also conducted the search update specific to physical activity in October 2015.

### Generic Review Methods

#### Identification of Studies for Review

A systematic search, common to all reviews, was undertaken in August 2012. Four sets of search terms were used related to: the population; study design (capturing observational, interventional and review articles); outcome; and exclusion of clinical populations. An extensive scoping phase was conducted prior to implementing the full search to maximize sensitivity and specificity of included papers. This involved contacting experts in the field and identifying key publications to be included for each behaviour, with searches run to ensure that these publications were captured. An electronic search was conducted in eight databases [MEDLINE, Embase (via OVID), CINAHL, PsycINFO (via EBSCO), Web of Knowledge (via Thomson Reuters), British Nursing Index (BNI), Applied Social Sciences Index and Abstracts (ASSIA) and Sociological Abstracts (via ProQuest)]. Citations were downloaded into Endnote citation management software (Thomson Reuters, Philadelphia, PA, USA). Included papers were searched for additional relevant publications, as were relevant reviews. No language restrictions were placed on the search, but articles were limited to published full texts. An updated search was conducted in October 2015 to capture studies with outcomes relating to physical activity only, published in the interim period (Electronic Supplementary Material Table S1).

#### Study Selection

In 2012, two batches of 500 titles and abstracts were screened for inclusion by the review leads (KH, VP, COM) and checked for fidelity by a fourth reviewer (CS). With less than a 5% discrepancy, each reviewer subsequently screened approximately 12,000 papers individually. For quality control, two random 5% samples (total *n* = 3600) were double screened by two additional reviewers (RL and EvS). All full texts were obtained and distributed for the behaviour-specific reviews to progress in parallel. Additional texts retrieved in 2015 were screened by KH and a subsample (15%) reviewed by EvS.

### Methods for Physical Activity Review

#### Inclusion/Exclusion Criteria

Articles were included if (a) they reported results from a longitudinal observational study, randomized controlled trial (RCT) or controlled trial (CT), (b) quantified a within-child change in physical activity behaviour (as primary/second outcome in interventions) and (c) assessed at least one potential determinant of change. Children had to be aged between 0 and 6 years at baseline, and studies assessing physical activity using objective or subjective measures were included. Exclusion criteria included: (a) clinical populations (e.g. children who were malnourished, had asthma, cerebral palsy, cystic fibrosis, autism, etc.), (b) non-human studies, (c) quantitative cross-sectional studies, (d) qualitative studies, and (e) laboratory-based studies (e.g. validation studies).

#### Quality Assessment

For descriptive purposes, a quality appraisal of each of the included studies was conducted focusing on internal and external validity using assessment criteria adapted from those used previously [34, 35] (Electronic Supplementary Material Table S2). Criteria included: sample representativeness, size and retention, use of objective exposure and outcomes measures, appropriateness of analysis strategy, and randomisation method for RCTs. Scores out of 6 (or 7 for RCTs) were allocated and categorised accordingly (high quality: ≥5; medium: 3–4; low: 1–2).

#### Data Extraction

All full texts identified for inclusion were read by KH, and double screened for inclusion by EvS. For relevant papers, data were extracted using a standardized form. Data extracted included fırst author; publication year; country; study design, setting and population; and baseline descriptive characteristics. Data were also extracted about physical activity measurement and outcome; potential determinants; method of analysis; duration of follow-up; loss to follow-up; and results. All outcome measures used in prospective and interventional studies [e.g. percentage time or minutes spent at differing activity intensities (i.e. light (LPA), moderate (MPA), vigorous (VPA), moderate to vigorous (MVPA) or total activity (LMVPA)] were extracted. However, in some studies, activity was only assessed during specific periods (i.e. at weekends, during recess). In an attempt to standardise findings across studies, where more than one physical activity outcome was reported, we report total physical activity/counts per epoch (given current guidelines for young children’s activity [19, 20]), followed by MVPA, LPA and MPA/VPA. For interventional studies, each of the described elements targeted in the intervention (e.g. parental knowledge, parental modelling) were extracted as potential determinants of change in physical activity. For each determinant, the smallest included sub-sample was considered for extraction (e.g. if stratified by sex). Where results were stratified by specific times of the day, results for the largest time periods were reviewed and extracted. For longitudinal studies, the latest data available before the children were 6 years old were included; where two or more papers reported on the same study sample, both were included if they reported determinants associated with different outcome measures. For interventional studies, we assessed the difference in physical activity between control and intervention groups over time to classify determinants, as this provided evidence of factors targeted in interventions (i.e. determinants) which were associated with change. Where possible, results of multivariable rather than univariable models were included.

### Data Synthesis

Narrative data synthesis was undertaken for all studies. Due to the heterogeneous nature of included quantitative studies and the physical activity outcomes used, meta-analysis was not appropriate. Each extracted determinant was scored based on direction and strength of evidence: ‘−’ significant decrease in physical activity; ‘0’ no significant association/effect or ‘+’ significant increase in physical activity. Evidence from cohort and interventional studies was weighted equally, as both provide prospective determinants of change in physical activity behaviour. As per previous reviews [16, 17, 36], consistency across studies for any given determinant was then summarized according to the following metric: ‘0’ (no association) if supported by 0–33% of individual studies; ‘?’ (indeterminate/possible) if supported by 34–59%; and ‘+’ or ‘−’ if supported by 60–100%. Where four or more studies reported on a potential determinant, double indicators were used (e.g. ‘00’, ‘??’, ‘++’ and ‘−−’) to indicate greater levels of evidence and therefore confidence in findings. Determinants, study score and consistency across studies were then presented according to the SEM (individual, interpersonal, organisational, community and policy) [17, 36].

## Results

**Fig. 1:**
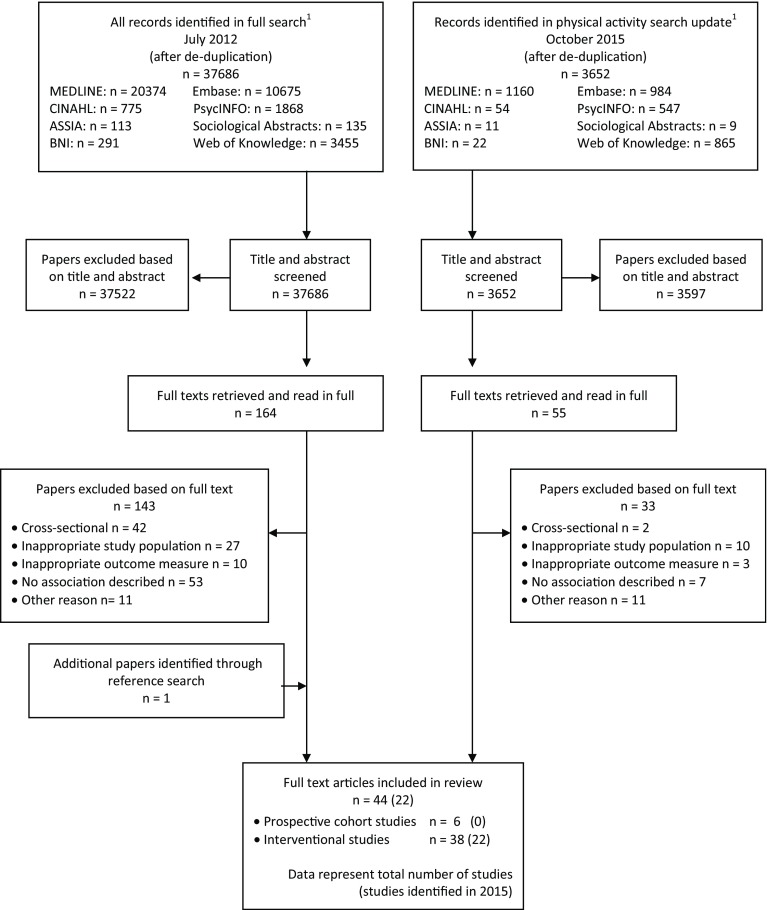
Flowchart outlining identification of papers for inclusion. *ASSIA* Applied Social Science Index and Abstracts, *BNI* British Nursing Index. ^1^Full search conducted including terms for all health behaviours (i.e. diet, physical activity), physical activity search update included terms for physical activity behaviours only

A total of 37,686 (full review) and 3652 (physical activity-specific update) references were retrieved in 2012 and 2015 respectively, of which 220 were read in full and 44 papers included for review (representing 42 study samples: four prospective cohort and 38 interventional studies, see Fig. 1). A descriptive summary of the included study samples is presented in Table 1; study-specific information is provided in Tables 2 and 3.

**Table 1:** Characteristics of included papers^a^

Sample characteristic	References	Total number of papers (%)
Study design		
Prospective	[60–62, 77–79]	6 (14)
Interventional	[11, 37–58, 64–67, 69–76, 81, 83, 84]	38 (84)
Total sample size		
<100	[37–40, 42, 44, 51, 53, 58, 69, 73, 76, 79, 81]	15 (34)
101–199	[41, 43, 56, 60–62, 65, 67, 70, 73, 77]	11 (25)
200–299	[45, 48, 54, 66, 74, 78]	6 (14)
300–399	[11, 47, 50, 55, 84]	5 (11)
400–499	[64, 72, 75]	3 (7)
500+	[46, 49, 57, 71]	4 (9)
Method of physical activity measurement		
Objective	[37–50, 53–56, 61, 62, 64–67, 73–76, 78, 79, 81, 83]	33 (77)
Subjective	[11, 51, 55, 57, 58, 60, 69–71, 77, 84]	11 (23)
Continent		
Australasia	[48, 51, 60–62, 78, 83]	8 (18)
Europe	[39, 41, 43–45, 49, 50, 64, 65, 75, 77, 79]	12 (27)
North America	[11, 37, 38, 40, 42, 46, 47, 52–58, 66, 67, 69, 71–74, 76, 81]	24 (55)
High quality (≥5^b^)		
Prospective	[78]	1 (4)
Interventional	[11, 40, 41, 43–48, 50, 52–56, 64, 65, 67, 72–76, 78, 83, 84]	26 (59)

^a^A total of 44 papers were included, describing 42 prospective and interventional studies

^b^Prospective studies scored out of 6, intervention studies scored out of 7

**Table 2:** Summary of studies included to assess determinants of PA levels in young children: study design, samples and setting

References	Study design/name	Population	Age at start (mean ± SD and/or range)	Setting
Prospective studies				
Ball et al. (2009), Cleland et al. (2008), Cleland et al. (2011) [60–62] Australia	Prospective cohort—CLAN	19 public elementary schools *n* = 168 (stratified by low/med/high SES)	5–6 y	Schools
Reilly et al. (2004) [79] UK	Prospective cohort—SPARKLE	Community level stratification *n* = 72 (51% M)	3.7 ± 0.5 y	Community
Saakslahti et al. (2004) [77] Finland	Prospective cohort	Cohort of children *n* = 155 (53% M)	4–7 y	Study subsample
Taylor et al. (2008) [78] New Zealand	Prospective cohort—FLAME	Population-based *n* = 244 (56% M; 86% W, 11% Maori, 3% PI; higher SES)	2.96–3.15	Birth cohort
intervention studies				
Alhassan et al. (2007) [40] USA	Pre-post; quasi-randomised	1 Low-income preschool *n* = 32 (63% M, predominantly Latino)	C: 3.59 ± 0.5 I: 3.89 ± 0.5	Headstart
Alhassan et al. (2012) [52] USA	Pre-post; quasi-randomised	2 preschools *n* = 78 (49% M; 39% AA, 61% H; 65% single-family homes)	C: 4.1 ± 0.6 I: 4.5 ± 0.6	Preschools
Alhassan et al. (2013) [53] USA	RCT—SPARK	2 preschools *n* (baseline) = 75; *n* (follow-up) = 67 (57% M)	2.9–5 y	Preschools
Annesi et al. (2013) [54] USA	cRCT—Start for Life	32 classrooms *n* = 275 (44% M; predominantly AA)	3.5–5.6 y (4.6 ± 0.5 y)	YMCA preschools
Annesi et al. (2013) [47] USA	cRCT—Start for Life	19 classrooms *n* = 338 (46% M; lower/lower–middle class; 92% AA)	C: 4.7±0.3 I: 4.6±0.6	YMCA preschools
Annesi et al. (2013) [46] USA	cRCT—Start for Life	26 classrooms *n* = 885 (46% M; lower/lower–middle class; 92% AA)	3.5–5.6 y (4.4 ± 0.5 y)	YMCA preschools
Bellows et al. (2013) [67] USA	RCT—The Food Friends: Get Movin’ with Mighty Moves	8 lower income Headstart centres *n* = 201 (55% M; 59% H, 32% W, 9% O)	I: 53.0 ± 6.8 mo C: 51.5 ± 6.6 mo	Headstart centres
Bonvin et al. (2013) [50] Switzerland	RCT—Youp’là Bouge	58 childcare centres *n* = 388 (50% M; 18% low educated parents; 58% migrant parents)	I: 3.4 ± 0.6 y C: 3.3 ± 0.6 y	Childcare centres in 3 French-speaking Cantons
Cardon et al. (2009) [41] Belgium	RCT	40 preschools *n* = 583 (52% M)	5.3 ± 0.4 y	Public preschools
Cottrell et al. (2005) [42] USA	RCT—CARDIAC-Kinder	29 preschools *n* (baseline) = 203 (49% M; 93% W) *n* (follow-up) = 50	5 ± 0.47 y	Preschools
Davis et al. (2013) [69] USA	Pilot intervention	Teen mothers, *n* = 60 (61% M; 73% AA; 16% W; 7% NA; 4% O)	0–53 mo (15.7 ± 13.4)	Child development programme
Davison et al. (2013) [81] USA	Pilot intervention	5 Headstart centres *n* (baseline) = 117 (45% M; 68% W; 22% AA; 6% non-H; 4% O) *n* (follow-up) = 57	3.59 ± 1.01 y	Headstart centres
De Bock et al. (2013) [49] Germany	cRCT	37 preschools *n* (baseline) = 809 (52% M; low income: 25%, middle income: 55%) *n* (follow-up) = 467	5.05 y	Preschools
De Coen et al. (2012) [71] Belgium	cRCT “Prevention of overweight among pre-school and school children (POP)”	31 schools across high, medium and low SES *n* = 1589 at baseline (I: 1032; C:557) *n* = 694 at 2 year (I: 396 C: 298)	4.95 ± 1.31 y	Pre-primary and primary schools
De Craemer et al. (2014) [64] Belgium	cRCT—Toybox	27 kindergartens in Flanders *n* = 472 (55% M)	4.43 ± 0.55 y	Kindergartens
Elder et al. (2014) [72] USA	RCT “MOVE/Me Muevo”	30 sites *n* = 541 (45% M; 41% H)	6.6 ± 0.7 y	Recreation centres
Eliakim et al. (2007) [65] Israel	RCT	4 preschools *n* = 101 (55% M; upper middle class)	5.5 y	Preschools
Engelen et al. (2013) [48] Australia	cRCT	12 schools *n* = 221 (54% M; ICSEA: 980–1170)	6.0 ± 0.6 y	Catholic primary schools
Fitzgibbon et al. (2005) [11] USA	cRCT—Hip-Hop to Health Jr	12 Headstart centres *n* = 409 (50% M; I: 99% AA, 1% O; C: 80.7% AA, 12.7% H, 6.6% O)	I: 48.6 ± 7.6 mo; C: 50.8 ± 6.4 mo	Headstart centres
Fitzgibbon et al. (2006) [55] USA	cRCT—Hip-Hop to Health Jr	12 Headstart centres *n* = 293 (50% M; I: 15.8% AA, 73.3% H, 10.9% O; C: 6.5% AA, 89.4% H, 4.0% O)	I: 50.8 ± 7.3 mo; C: 51.0 ± 7.0 mo	Headstart centres
Fitzgibbon et al. (2011) [56] USA	cRCT—Hip-Hop to Health	18 Headstart centres *n* (baseline) = 223 (44% M; I: 97% AA, 1% H, 2% O; C: 91% AA, 5% H, 4% O) *n* (follow-up) = 190	I: 50.7 ± 6.8 mo C: 51.9 ± 6.3 mo	Headstart programmes
Fitzgibbon et al. (2013) [73] USA	cRCT—Hip-Hop to Health	4 centres *n* (baseline) = 146 (50% M; 94% H; 2% AA; 4% O) *n* (follow-up) = 123	54.2 ± 5.0 mo	Early childhood education programmes
Hannon and Brown (2008) [37] USA	Pre-post intervention	1 centre *n* = 64 (47% M; predominantly W)	3.9 ± 0.8 y	Preschool
Jones et al. (2011) [83] Australia	Non-randomised pilot “Time 2b Healthy”	Overweight preschool children and parents; *n* (baseline) = 46 (~80% parents had degree/tech trade cert) *n* (follow-up) = 40	2–5 y	Home based
Jones et al. (2011) [51] Australia	Pilot RCT “Jump Start”	2 low-income centres *n* = 97	4.1 y	Preschools
Klohe-Lehman et al. (2007) [58] USA	Non-randomised trial	Low-income, overweight or obese mothers n=235 (62.6% H)	1–3 y (mean 2.1 y)	Public health clinics / groups
O’Dwyer et al. (2012) [44] UK	cRCT	8 preschools *n* = 79 (52% M)	<5 y	Home based
O’Dwyer et al. (2013) [45] UK	cRCT	12 centres *n* = 240 (56% M; I: 84.3% W; C:75.3 W)	3.7 ± 0.6 y	Sure Start centres
Ostbye et al. (2012) [74] USA	RCT—KAN-DO	Patient records *n* = 400 (56% M)	3.1 ± 1.0 y	Healthcare
Puder et al. (2011) [75] Switzerland	cRCT - Ballerbina	40 centres *n* = 652 (50% M; 40% speak foreign language at home; 62% with 2 educated parents)	5.1 ± 0.7 y	Preschools
Stark et al. (2011) [76] USA	Pilot RCT “LAUNCH”	Children with BMI ≥ 95th% and 1+ overweight parent *n* = 15	2–5 y (mean 4.7 ± 1.1 y)	Home and clinics
Stratton and Mullan (2005) [39] UK	Pilot RCT	4 schools *n* = 54 (46% M; low SES areas)	4–7 y	Primary schools
Trost et al. (2008) [38] USA	RCT—Move and Learn	1 centre *n* = 42 (55% M; 23.7% with high school diploma)	4.1 ± 0.7 y	Childcare centre
van Cauwenerghe et al. (2012) [43] Belgium	Pilot intervention	4 preschools *n* = 128 (55% M)	4–6 y	Preschools
Verbestel et al. (2013) [70] Belgium	Pilot RCT	60 centres *n* = 203 (54% M)	15.5 ± mo	Daycare centres
Wen et al. (2012, 2015) [84, 106] Australia	Non-randomised intervention “Healthy Beginnings”	Low-income mothers *n* = 465 (11% spoke language other than English at home)	From birth	WIC sites
Whaley et al. (2010) [57] USA	Non-randomised trial “Child health and intervention research project” (CHIRP)	Low-income mothers *n* (baseline) = 821, (94% H; 50% mothers of M); *n* (follow-up) = 589	1–5 y (mean 23 ± 9.2 mo)	WIC sites
Yin et al. (2012) [66] USA	Pre-post intervention	4 centres *n* = 390 (59% M; 62% normal weight; predominantly H)	4.1 ± 0.56 y	Headstart centres

*PA* physical activity, *RCT* randomised controlled trial, *cRCT* cluster randomised controlled trial, *CLAN* Children Living in Active Neighbourhoods, *KAN-DO* Kids and Adults Now – Defeat Obesity!, *SPARK* Sports, Play, and Active Recreation for Kids, *SPARKLE* Study of Preschool Activity, Lifestyle and Energetics, *LAUNCH* Learning about Activity and Understanding Nutrition for Child Health, *FLAME* Family Lifestyle, Activity, Movement, and Eating, *CARDIAC* Coronary Artery Risk Detection In Appalachian Communities, *ICSEA* Index of Community Socio-Educational Advantage, *I* intervention group, *C* control group, *SES* socio-economic status, *M* male, *W* White, *AA* African American, *H* Hispanic, *NA* Native American, *O* other racial group, *PI* Pacific Islander, *BMI* body mass index, *cert* certificate, *med* medium, *y* years, *mo* months, *WIC* women, infants and children, *SD* standard deviation

**Table 3:** Summary of studies included to assess determinants of PA levels in young children: interventions (type, provider, duration), targeted determinants, outcomes, measures, effects and quality scores

References	Intervention and provider	Targeted determinants [theoretical model]	Intervention duration (or follow-up)	Outcome	Measure	Effect	Quality score^a^
Prospective studies							
Ball et al. (2009), Cleland et al. (2008), Cleland et al. (2011) [60–62] Australia	N/A	Child: sex Parents: behaviour, psychosocial Temporal: time of day, week, season	Up to 5 y	Ball: Change in cpm Cleland: change in MVPA	Accelerometer	cpm: 0 MVPA: + (for limited determinants)	4
Reilly et al. (2004) [79] UK	N/A	Children: sex	1 y	Change in total PA	Accelerometer	TEE: +	3
Saakslahti et al. (2004) [77] Finland	N/A	Children: sex	2 y	Change in time spent in high intensity PA	Questionnaire	Change in high intensity PA: 0	2
Taylor et al. (2008) [78] New Zealand	N/A	Children: sex	3 y	Change in MVPA	Accelerometer	MVPA: 0	5
Intervention studies							
Alhassan et al. (2007) [40] USA	60 min of additional recess time per day, divided into two 30-min blocks (one in the morning and one in the afternoon) [vs. usual recess time]	Preschool: additional PA time [no theory identified]	2 d	Change in cpm	Accelerometer	cpm: 0	2
Alhassan et al. (2012) [52] USA	Delivered for 30 min/d, five d/wk for 6 mo during morning gross motor playtime. Motor skill curriculum: 30 individual lesson, with one skill per lesson, e.g. 5 min of low-intensity musical activity, 20 min of motor skills, 5 min of reinforcement	Multi-level, including Children: motor skills Preschool: provider training (8 h) [no theory identified]	6 mo	Change in % time MVPA	Accelerometer	% MVPA: 0	5
Alhassan et al. (2013) [53] USA	Both I&C given 30 min of additional outdoor playtime for 3 d/wk for 4 weeks I: Providers delivered 12 sessions structured activity programme to increase MVPA	Preschool: provider training (8 h), additional PA time [no theory identified]	4 wk	Change in minutes % time in MVPA	Accelerometer	% MVPA: 0	6
Annesi et al. (2013) [54] USA	Provider-delivered structured activity including gross motor skills and behavioural skills training (30 min/d)	Multi-level, including Children: motor, behavioural skills Preschool: provider training (4 h) [social cognitive and self-efficacy theory]	8 wk	Change in MVPA	Accelerometer	MVPA: +	6
Annesi et al. (2013) [47] USA	Provider-delivered structured activity including gross motor skills and behavioural skills training for 30 min/d	Multi-level, including Children: motor, behavioural skills Preschool: provider training (4 h) [social cognitive and self-efficacy theory]	8 wk	Change in MVPA	Accelerometer	MVPA: +	6
Annesi et al. (2013) [46] USA	Provider-delivered structured activity including gross motor skills and behavioural skills training for 30 min/d	Multi-level, including Children: motor, behavioural skills Preschool: provider training (4 h) [social cognitive and self-efficacy theory]	8 wk (9 mo)	Change in MVPA	Accelerometer	MVPA: +	6
Bellows et al. (2013) [67] USA	Provider led skills-based 72-lesson programme (4 d/wk for 15–20 min, for 18 wk). Focus on stability, locomotor or manipulation, then skill patterns. Use of Food Friends characters and other materials to support lessons. Materials sent home	Multi-level, including Children: motor, behavioural skills Parents: knowledge Preschool: provider training (8 h) [no theory identified]	18 wk	Change in mean daily steps (wk/e and wk/d) (2o)	Pedometer	Steps: 0	6
Bonvin et al. (2013) [50] Switzerland	Multi-component PA programme, delivered to children and parents via providers in preschools. Preschools left to implement PA programme according to their own needs	Multi-level, including Children: skills, knowledge Parents: encouraged engagement, knowledge Preschool: provider training/support; changes in built environment ($1500) [no theory identified]	9 mo	Change in cpm, MVPA (2o)	Accelerometer	cpm: 0	6
Cardon et al. (2009) [41] Belgium	Factorial design: 1: play equipment provided (150 children); 2: markings painted on the playground (161); 3: play equipment and markings provided (161)	Preschool: changes in environment [no theory identified]	6 mo	Change in cpe	Accelerometer	cpe: 0	6
Cottrell et al. (2005) [42] USA	Children received 2 pedometers—one for themselves and one for a parent (vs. one for child in C group) and step log. Also received information building on activity and diet recommendations	Multi-level, including Children: monitoring, knowledge Parents: monitoring, knowledge [no theory identified]	4 wk	Change in weekly average steps	Pedometer	Weekly steps: + (week 4)	2
Davis et al. (2013) [69] USA	In-home intervention focusing on nutrition and activity: 3 sessions for mother, 3 focused on child. Providing information, and including behavioural topics such as goal setting, tracking, social support	Multi-level, incl Parents: knowledge, monitoring, goal setting, social support Organisational: facilitator training (4 h) [no theory identified]	3 mo	PA in past week; PA in typical week	Questionnaire	Change in typical week: +	2
Davison et al. (2013) [81] USA	Multi-component intervention delivered through Head Start centres, including health communication campaign, BMI letters, family nutrition counselling, parent skill sessions, and similar programme for children	Multi-level, including Children: encouragement, knowledge Parents: skills training, knowledge Community: awareness [family ecological model]	6 mo	Change in min/ h LPA, MPA (2o)	Accelerometer	LPA: + MPA: 0	4
De Bock et al. (2013) [49] Germany	Augmentation of 6 mo State programme (+3 mo) to motivate parents to promote children’s PA. Introductory video and project ideas, with external gym trainers provided for I school to coordinate parent activities. Initial workshop followed by teambuilding and implementation of projects as regular activities	Multi-level, including Parents: motivation, skills training, knowledge Preschool: additional providers, provider training [participatory intervention approach]	9 mo	Change in cp15s	Accelerometer	cp15: +	4
De Coen et al. (2012) [71] Belgium	Health promotion programme with child at centre, including range of potential carers/those influencing activity (family, friends, schools, community, stakeholders, local policy and media)	Multi-level, including Child: knowledge Parents: knowledge School: knowledge, Policies change Community: knowledge [socio-ecological theory]	2 school y (09/08–04/10)	Change in hours of sports club and after-school activity participatio*n* (2o)	Questionnaire	Sport: 0 After-school: 0	4
De Craemer et al. (2014) [64] Belgium	Health promotion programme with children within centres, PA component implemented in wk 5–8, with 2-wk repetition period in wk 19–20. Materials provided to be used for minimum of 1 h/wk. Newsletters (with key messages on PA) and tip-cards sent home	Multi-level, including Child: knowledge Parents: knowledge School: curriculum materials, provider knowledge, provider training [PRECEDE-PROCEED, intervention mapping]	24 wk	Change in total PA on wk/d	Accelerometer	Total PA: 0	5
Elder et al. (2014) [72] USA	Tailored to the family’s needs to target physical and social aspects of the home environment. Initial call; 1.5-h group workshop and 1-h home visit. Tip sheets to promote healthy eating and physical activity to their children. PA: (1) increase the amount of MVPA to 60 min/d; (2) increase PA opportunities; (3) increase the variety of fun, developmentally/ culturally appropriate PA	Multi-level, including Parents: knowledge, social support Centre: facilitator training Community: awareness [no theory identified]	2 y	Change in total active time	Accelerometer	Total PA: +	6
Eliakim et al. (2007) [65] Israel	Health promotion programme (4 mo) PA: 45 min/d of exercise (6 d/wk), twice co-ordinated by a professional youth coach; sessions split into 3 × 15 min sessions. Training: duration, intensity, co-ordination and flexibility plus reduce sedentary time and increase after school PA	Multi-level, including Children: skills training Preschool: additional PA time; additional providers [no theory identified]	14 wk	Change in total daily steps	Pedometers	Steps: +	5
Engelen et al. (2013) [48] Australia	Playground-based intervention introducing portable equipment (13 w) and a 2-h teacher-parent intervention exploring risk administered (2–3 wk post-playground intervention initiation)	Multi-level, including Parents: knowledge School environment: change in environment, provider knowledge [no theory identified]	13 wk	Change in cpm, MVPA daily	Accelerometer	cpm: 0	5
Fitzgibbon et al. (2005) [11] USA	Health promotion programme. 40-min sessions 3/wk, covering a different theme: 20 min of introducing health promoting topic and 20 min of PA, including the use of colourful puppets. Parents received a weekly newsletter, covering healthy eating, PA and a homework task (5 min daily or 15 min one off)	Multi-level, including Children: knowledge, Parents: knowledge Preschool: additional PA time, curriculum materials [social cognitive theory]	14 wk	Change in PA (2o)	Parental self-report: frequency/intensity (% >7×/w, Borg scale)	Frequency: 0 Intensity: 0	5
Fitzgibbon et al. (2006) [55] USA	Health promotion programme. 40-min sessions 3/wk, covering a different theme: 20 min on nutrition (food pyramid) and 20-min aerobic PA. Parents received 12 homework assignments during the 14-week intervention (with incentive)	Multi-level, including Children: knowledge Parents: knowledge Preschool: additional PA time, curriculum materials [social cognitive theory]	14 wk [1 and 2 y post intervention]	Change in PA (2o)	Parental self-report frequency/ intensity (% >7× /wk, Borg scale)	Frequency: 0 Intensity: 0	5
Fitzgibbon et al. (2011) [56] USA	Health promotion programme. 40-min 2/wk (optional 3rd). 20 min on nutrition (food pyramid) and 20 min aerobic PA, incorporating musical CD for teachers. Parental homework: 6 areas related to cultural practices and beliefs: food, family, music, community, social roles, and relationships	Multi-level, including Children: knowledge Parents: knowledge Preschool: additional PA time, curriculum materials [social cognitive and self-determination theory]	14 wk	Change in MVPA (min/d) and counts/min (2o)	Accelerometer	cpm: 0 MVPA: +	5
Fitzgibbon et al. (2013) [73] USA	Health promotion programme. 40-min sessions 3/wk, covering a different theme: 20 min on nutrition (food pyramid) and 20 min aerobic PA. Parents also participated in a 30 min exercise session. Parent component: 6 × 90 min/wk (60 min of interactive instruction on diet and PA, 30 min MVPA classes) + newsletters for a lower-income, Hispanic population	Multi-level, including Children: knowledge Parents: knowledge, PA classes Preschool: additional PA time, curriculum materials [social cognitive theory]	14 wk	Change in cpm/MVPA (2o)	Accelerometer	cpm: 0 MVPA: 0	5
Hannon and Brown (2008) [37] USA	Introduction of age-appropriate portable toys in playground on intervention days, including hurdles, hoops, tunnels, balance beams, balls	Preschool: change in environment [no theory identified]	5 d pre/post	Change in % MPA/VPA outdoor play/d	Accelerometer and OSRAC-P	MPA: + VPA: +	5
Jones et al. (2011) [83] Australia	Interactive online parental education and discussion forums (5 modules, each module lasting 2 weeks) to promote healthy lifestyles in overweight preschool-aged children	Parents: knowledge, parenting skills, social support [aligned to Healthy Eating and Physical Activity (Australian Government)]	10 wk	Change in PA behaviours	Parental self-report	Child doing regular PA: +	2
Jones et al. (2011) [51] Australia	Structured lessons 3× wk for 20 wk: 20-min lesson focused on one fundamental movement skill. Each skill comprised a number of components, e.g. running had four. Practice through fun activities and games. Unstructured activities facilitated in the afternoons for practice with equipment	Multi-level, including Children: motor skills Preschool: provider training (2 h) [no theory identified]	20 wk	Change in cpm	Accelerometer	cpm: 0	3
Klohe-Lehman et al. (2007) [58] USA	Weight loss intervention for mothers (8× weekly 2-h classes: 15-min weigh-in, 1.25-h discussion and activities, 30-min exercise). Delivered by registered dieticians	Multilevel, including Parents (mothers) knowledge, modelling, parenting skills Home environment opportunities for PA [social cognitive theory]	8 wk	Change in PA (mother and child)	Toddler Behavior Assessment Questionnaire (TBAQ)	Change PA: +	3
O’Dwyer et al. (2012) [44] UK	5 sessions (70 min: 10 min registration, 60-min delivery) 1 every 2 wk. Parents and children separate for first 20 min, 40 min spent together as a group. Active play for children delivered by play workers, educational workshop for parents. Parents monitored PA at home with logbook, linked to a reward system. Text message reinforcement	Multilevel, including Children: additional PA time Parents (mothers) knowledge, modelling, monitoring [socio-ecological theory]	10 wk	Change in total weekday PA	Accelerometer	Wk/d PA: +	6
O’Dwyer et al. (2013) [45] UK	Active play intervention (60 min 1/w) with staff training to deliver active curriculum. 2-2-2 format: 2 wk practitioner, 2 wk co-delivery, 2 wk teacher, with practitioner facilitating. Resource pack provided to preschools along with user manual and exemplar lesson plans and promotion poster	Preschool: staff training, additional staff [socio-ecological theory]	6 wk [and 6 mo]	Change in MVPA	Accelerometer	MVPA: 0	6
Ostbye et al. (2012) [74] USA	8 monthly mailed interactive kits; 20–30 min motivational interviewing coaching session via phone. Kits included activities and incentives. Targeted healthy weight via instruction in parenting styles and skills, techniques for stress management and education. One semi-structured group session also included: a healthy meal and free childcare were provided	Multi-level, including Children: monitoring Parents: knowledge, social support, monitoring [socio-cognitive theory]	8 mo	Minutes of MVPA per day	Accelerometer	MVPA: 0	6
Puder et al. (2011) [75] Switzerland	Multidimensional culturally tailored lifestyle intervention, with workshops, lessons, home activities, offers of extracurricular activities and adaption of the built environment. Teacher training (2 workshops); PA programme (4 × 45 min/wk with CD); Activity cards to take home; 1 meeting of parents and teachers	Multi-level, including Children: skills and fitness Parents: knowledge, participation, social support Preschool: provider training, change in built environment, social support, additional PA time, curriculum materials [socio-ecological theory]	11 mo	Change in PA (2o)	Accelerometer	Accelerometer: 0	6
Stark et al. (2011) [76] USA	Enhanced pediatric counselling. Intervention and maintenance: 12 wkly and 2 wkly sessions (group-based clinic parent-child sessions or individual home visits. Children and parents given pedometers and goals of 5000 and 10,000 steps/d, as feedback. Delivered by paediatricians and psychologists at parent-groups, child-groups and home visits	Multi-level, including Children: knowledge, goals Parents: knowledge, parenting skills, parental modelling, goal setting [social cognitive theory]	36 wk [6 and 12 mo]	Change in MPA, VPA (2o)	Accelerometer	MPA: 0 VPA: 0	5
Stratton and Mullan (2005) [39] UK	Playground markings; painted in bright fluorescent colours according to school preference: e.g. castles, dragons, clock faces, mazes, fun trails, dens, hopscotch, letter squares, snakes and ladders	Preschool: change in environment [no theory identified]	6 mo	Heart rate; Play time in MPA, VPA	Telemeter	MPA: 0 VPA: +	4
Trost et al. (2008) [38] USA	PA opportunities integrated into all aspects of the preschool curriculum Teachers were required to include 2 Move and Learn curriculum activities lasting 10 min or longer in each 2.5-h session (4/d). Activities were typically repeated several times throughout the week	Preschool: Additional PA time, Provider training [no theory identified]	10 wk	Change in MVPA	Accelerometer & OSRAP	MVPA (w5–8): +	2
van Cauwenerghe et al. (2012) [43] Belgium	Lowering playground density	Preschool: change in environment [no theory identified]	1 wk	Change in daily LMVPA	Accelerometer	Daily LMVPA: 0	5
Verbestel et al. (2013) [70] Belgium	Family-based healthy lifestyle intervention: improve diet, PA levels and decrease screen-time. Two components: (1) guidelines and tips on poster with stickers (every 2 mo, along with additional tip sheet) (2) a tailored feedback form for parents about their children’s activity- and dietary-related behaviours	Multi-level, including Children: goal setting Parents: knowledge, goal setting, monitoring [information processing; elaboration likelihood model; precaution-adoption-process model]	1 y	Time spent in PA	Question	PA time: 0	4
Wen et al. (2012, 2015) [84, 106] Australia	8 home visits from nurses delivering staged home-based intervention: one antenatal visit, then at 1, 3, 5, 9, 12, 18, and 24 mo after birth, with ongoing telephone support. 1-h visits: monitoring the parent-child feeding interaction and practice, and behaviours promoting physical activity/inactivity in the child. Needs identified with checklist and fed back. Problem-solving, individualized information kit and phone feedback provided	Parents: parenting skills, social support [no theory identified]	2 y, 5 y post intervention	Outdoor play ≥120 min/d	Questionnaire	Outdoor play: 0	5
Whaley et al. (2010) [57] USA	Enhanced questionnaire and 1-2-1 MI with mothers to discuss one of 6 health behaviour topics [PA: getting up and moving more] at their 6-mo WIC recertification appointments. Delivered by WIC staff using motivational interviewing techniques	Parents motivation, social support [trans-theoretical model]	1 y: 6 and 12 mo	Engaging in >60 min of PA (d/w)	Questionnaire	Engaging in PA: 0	3
Yin et al. (2012) [66] USA	Home-, centre- and curriculum-based intervention for diet and physical activity. Factorial design (centre, home, centre and home). Centre-based including staff training, curriculum resources and 60 min structured and free play/d. Home-based peer-led parent obesity education, homework, family support and monitoring for PA	Multi-level, including Children: motor skills Parents: knowledge, social support, monitoring Preschool: provider training, additional PA time [early child development and systems approach]	18 wk	Steps/min in outdoor play	Pedometers	Steps/min in outdoor play: +	4

*PA* physical activity, *CLAN* Children Living in Active Neighbourhoods, *KAN-DO* Kids and Adults Now – Defeat Obesity!, *SPARK* Sports, Play, and Active Recreation for Kids, *SPARKLE* Study of Preschool Activity, Lifestyle and Energetics, *LAUNCH* Learning about Activity and Understanding Nutrition for Child Health, *FLAME* Family Lifestyle, Activity, Movement, and Eating, *CARDIAC* Coronary Artery Risk Detection In Appalachian Communities, *PRECEDE-PROCEED* Predisposing, Reinforcing and Enabling Constructs in Educational Diagnosis and Evaluation - Policy, Regulatory, and Organizational Constructs in Educational and Environmental Development, *OSRAP* Observational System for Recording Activity in Preschools, *OSRAC-P* Observational System for Recording Physical Activity in Children-Preschool Version, *MI* motivational interviewing, *I* intervention group, *C* control group, *cpm* counts per minute, *cpe* counts per epoch, *cp15* counts per 15 s, *LPA* light physical activity, *MPA* moderate physical activity, *MVPA* moderate to vigorous physical activity, *VPA* vigorous physical activity, *LMVPA* total physical activity (i.e. light, moderate and vigorous physical activity), *TEE* total energy expenditure, *2o* measured as secondary outcome, *BMI* body mass index, *wk/e* weekend, *wk/d* weekday, *d* day, *h* hour, *wk* week, *y* years, *mo* months, *N/A* not applicable, *WIC* women, infants and children, + statistically significant positive effect of intervention, 0 no effect of intervention

^a^Out of 6 for prospective and 7 for interventional studies

### Summary of Study Characteristics

Study samples originated in the USA (*n* = 24), Australasia (*n* = 6) and Europe (*n* = 12); no papers were identified from developing nations, and all bar one was published after 2003. Of included studies, 15 (34%; 13 interventional, two prospective) had a final sample size greater than 250 children, and most included similar numbers of boys and girls. Objective measures of physical activity were used in 34 (77%) papers (accelerometer: 27; pedometer: four; heart-rate/Actiheart: three), although those papers using proxy-report measures were also included (*n* = 10; one prospective, nine interventional). Interventions often targeted a number of behaviours, including diet and sedentary behaviour, but 18 (38%) specifically aimed to increase physical activity [37–54]. The measurement period (from baseline to last contact) was a median of 2.5 years (range 1–5 years) for prospective papers and 34.5 weeks (range: 1 day to 5 years post-intervention) for interventional papers. One prospective paper and 26 interventional papers (61%) were deemed to be of high quality (score ≥5), nine were of medium quality (score 3–4) and six were low quality (score of 2). Of the interventional studies, 28 (64%) randomised participants. Most study samples drew participants from White populations; some targeted lower socioeconomic or racial minority groups [11, 55–58]. A retention rate of ≥70% was reported in 20 papers (46%), and 27 interventional studies reported final analysis samples by study group, indicating similar levels of attrition.

### Overview of Prospective and Intervention Studies

A total of 44 potential determinants of change were reported (Table 4) across papers. The same cohort study [Children Living in Active Neighborhoods (CLAN) [59]] was described in three [60–62] of the six prospective papers. One paper describing this study contributed all 16 determinants identified across prospective studies in intrapersonal, interpersonal and temporal domains. This paper predominantly reported on determinants relating to parental influence on change in physical activity.

**Table 4:** Determinants assessed in prospective and interventional studies

Determinant	Association with change in physical activity			Studies showing positive association	Outcome
	−	0	+		
Intrapersonal (child)					
Sex (boys)				2/5	??
Questionnaire	[77]				
Total activity (counts per epoch)		[60]^b^	[79]		
MVPA		[78]	[61]^b^		
Motor/skill training^a^				5/10	??
Total activity (counts per epoch)		[50, 51, 75]			
Pedometer		[80]	[65, 66]		
MVPA		[52]	[46, 47, 54]		
Knowledge^a^				1/11	00
Questionnaire		[11, 55, 71]			
Total activity (counts per epoch)		[50, 56, 73, 75]			
Pedometer			[42]		
MVPA		[64, 76, 81]			
Goal setting^a^		[76]		0/1	0
Monitoring^a^				1/3	0
Questionnaire		[70]			
Pedometer			[42]		
MVPA		[82]			
Fitness^a^		[75]		0/1	0
Interpersonal					
Family demographics					
Maternal SES		[60]^b^		0/1	0
Sibling PA level		[61]^b^		0/1	0
Parental psychosocial					
Maternal reinforcement		[61]^b^		0/1	0
Paternal reinforcement		[61]^b^		0/1	0
Maternal role-modelling^a^				3/3	+
Questionnaire			[58]		
MVPA			[44 61 ^b^]		
Paternal role-modelling		[61]^b^		0/1	0
Parental role-modelling^a^				0/3	0
Questionnaire		[70]			
MVPA		[76, 82]			
Parental monitoring^a^				4/6	++
Questionnaire		[70]	[69]		
Total activity (counts per epoch)		[72]			
Pedometer			[42, 66]		
MVPA			[44]		
Parental motivation^a^				1/4	00
Questionnaire		[57]			
Total activity (counts per epoch)		[72]	[49]		
MVPA		[82]			
Parental goal setting^a^				2/4	??
Questionnaire			[69, 83]		
Total activity (counts per epoch)		[72]			
MVPA		[76]			
Parental knowledge^a^			,	7/22	00
Questionnaire		[11, 55, 70, 71]	[58, 69, 83]		
Total activity (counts per epoch)		[48, 50, 56, 72, 73, 75]	[49]		
Pedometer		[80]	[42, 66]		
MVPA		[64, 76, 81, 82]	[44]		
Parent skills^a^				2/7	00
Questionnaire		[84]	[83]		
Total activity (counts per epoch)		[51]	[49]		
MVPA		[76, 81, 82]			
Parental self efficacy^a^				1/4	00
Questionnaire		[57, 70]			
Pedometer			[66]		
MVPA		[82]			
Parental social support^a^				2/5	??
Questionnaire		[84]	[69, 83]		
Total activity (counts per epoch)		[72, 75]			
Parental behaviour					
Maternal co-participation		[61]^b^		0/1	0
Paternal co-participation		[61]^b^		0/1	0
Parental co-participation^a^		[75]		0/1	0
Siblings co-participation			[61]^b^	1/1	+
Family participation		[61]^b^		0/1	0
Maternal direct support		[61]^b^		0/1	0
Paternal direct support		[61]^b^		0/1	0
Opportunities for play^a^				2/2	+
Questionnaire			[58]		
MVPA			[44]		
Organisational					
Preschool environment					
Provider training^a^				8/16	??
Total activity (counts per epoch)		[50, 51, 72, 75]	[49]		
Pedometer		[80]	[66]		
MVPA		[45, 52, 64]	[38, 44, 46, 47, 53, 54]		
Provider knowledge^a^				0/2	0
Total activity (counts per epoch)		[48]			
MVPA		[64]			
Provider social support^a^		[75]		0/1	0
Additional providers^a^				2/3	+
Total activity (counts per epoch)			[49]		
Pedometer			[65]		
MVPA		[45]			
Increased active time^a^		[11, 55]		4/11	??
Questionnaire					
Total activity (counts per epoch)		[56, 73, 75]			
Pedometer			[65, 66]		
MVPA		[40, 45]	[38, 53]		
Structured physical activity^a^			[53]	1/1	+
Playground density (low)^a^			[43]	1/1	+
Playground markings^a^		[41]	[39]	1/2	0
Portable equipment^a^				1/5	00
Total activity (counts per epoch)		[41, 48, 50, 75]			
MVPA			[37]		
Curriculum materials^a^				2/11	00
Questionnaire		[11, 51, 71]			
Total activity (counts per epoch)		[50, 56, 70, 73]	[49]		
Pedometer		[80]			
MVPA		[64]	[53]		
Preschool policy change^a^		[71]		0/1	0
Centre monitoring/feedback^a^		[72]		0/1	0
Community					
Community awareness^a^				0/3	0
Total activity (counts per epoch)		[72]			
Pedometer		[71]			
MVPA		[81]			
Temporal					
Time of the day		[62]^b^		0/1	0
Time of the week		[62, 78, ]		0/2	0
Season		[62]^b^		0/1	0

For 1–3 studies: 0: 0–33% of papers support positive/negative association; ?: 34–59% support positive/negative association; +/ −: 60–100% support positive or negative association. For ≥4 studies: 00: 0–33% of papers support positive/negative association; ??: 34–59% support positive/negative association; ++/−−: 60–100% support positive or negative association

*SES* socio-economic status, *PA* physical activity, *MVPA* moderate-to-vigorous physical activity

^a^Interventional components

^b^Indicate prospective studies, all others are interventional studies

The 38 interventional studies targeted 28 potential (modifiable) determinants at intrapersonal (*n* = 6), interpersonal (*n* = 10), organisational (*n* = 10) and community levels (*n* = 1). No determinants at the policy level were identified across included studies. Of the 38 interventional studies, 27 (68%) were classified as multi-level [11, 42, 44, 46–48, 50–52, 54–56, 58, 63–76]; these most commonly targeted individual/interpersonal (i.e. children, parents, teachers) and organisational (i.e. preschool/ home environment) factors. Of these, 11 multi-level interventions (42%) effected a positive change in children’s physical activity [42, 44, 46, 47, 54, 58, 63, 65, 66, 69, 72], though no clear effective combinations of components emerged. Across all prospective studies, positive effect sizes were generally small, with increases of less than 10% in total activity or MVPA from relatively low baseline levels.

### Determinants Identified in Four or More Studies

Fourteen determinants were assessed in four or more studies. One, sex, was reported in five prospective papers [60, 61, 77–79] (from four study samples: the associations between sex and two different outcome measures were assessed within the same CLAN study sample). The remaining 13 determinants, reported four or more times, were all interventional components, including at the intrapersonal level: motor/skills training [46, 47, 50–52, 54, 65, 66, 75, 80] and child knowledge [11, 42, 50, 55, 56, 64, 71, 73, 75, 76, 81], and at the interpersonal level: parental monitoring [42, 44, 66, 69, 70, 72]; parental motivation [49, 57, 72, 82]; goal setting [69, 72, 76, 83]; parental knowledge [11, 42, 44, 48–50, 55, 56, 58, 64, 66, 69–73, 75, 76, 80–83]; general parental skills [49, 51, 76, 81–84]; parent self-efficacy [57, 66, 70, 82]; parental social support [69, 72, 75, 83, 84]; and provider training [38, 44–47, 49–54, 64, 66, 72, 75, 80]. Those determinants at the organisational level included: more physical activity opportunities [11, 38, 40, 45, 53, 55, 56, 65, 66, 73, 75]; use of portable equipment [37, 41, 48, 50, 75]; and supplying curriculum materials [11, 49, 50, 53, 55, 56, 64, 71, 73, 75, 80].

Of these 14 more frequently studied determinants, parental monitoring was consistently shown to be positively associated with change in young children’s physical activity across intensities, with four of six study samples reporting a positive association. Provider training was also positively associated with change in children’s MVPA in six of nine studies [38, 44, 46, 47, 53, 54], but showed no clear association with physical activity overall (positive association in 8/16 studies), suggesting that determinants may be intensity specific.

Five determinants, across the intra- and interpersonal domains, namely sex (positive association in 2/5 studies); motor skill training (5/10); parental goal setting (2/4); parental social support (2/5); and increased time for physical activity (usually within the care setting; 4/11) showed no consistent association with change in physical activity. In the case of sex, evidence from the CLAN study served to highlight how determinants may differ within the same sample depending on the outcome used and time of follow-up [i.e. no association with counts per epoch at first follow-up [60] but a positive association between (male) sex and MVPA at second follow-up [61]]. For motor skills training [46, 47, 54, 65, 66] and increased time for physical activity [38, 53, 65, 66] the majority of interventional studies that found a positive association with change in physical activity used objective measures.

The remaining seven determinants assessed in four or more studies, i.e. child knowledge (positive association in 2/12 studies), parental knowledge (7/22), parenting skills (2/7), parental motivation (1/4), parental self-efficacy (1/4), curriculum materials (2/11), and portable equipment (1/5), consistently showed no association with change in young children’s physical activity (i.e. >67% of studies reported no association).

### Determinants Identified in Fewer than Four Studies

Determinants assessed in three study samples in the intra-/interpersonal domains included child monitoring [42, 70, 82], parental role-modelling [70, 76, 82] and maternal role modelling [44, 58, 61], with only the latter shown to be positively associated with change in physical activity in all three studies (one using proxy-reported physical activity [58]). In the organisational domain, increasing the number of care providers within the childcare setting was found to be positively associated with change in two (out of three) interventional studies [49, 65]. Community awareness showed no association with change in children’s physical activity [71, 72, 81]. Positive associations with change in physical activity were also found for providing additional opportunities for play within the home (two studies) [44, 58] and sibling co-participation (one study) [61], and with structured physical activity [53] and lowering playground density [43] in one study each within the organisational domain.

## Discussion

### Main Findings

This review is the first to synthesise evidence from longitudinal studies relating to the determinants of change in physical activity in preschool-aged children. Forty-four determinants were identified; determinants at the interpersonal and organisational levels were most commonly evaluated. Fourteen determinants were identified in four or more quantitative studies: parental monitoring showed a consistently positive association with change in physical activity. Provider training was positively associated with change in MVPA, but showed no clear association with physical activity overall. Of the remaining 12 determinants, a further five showed no clear association, and seven were consistently not associated with change in children’s physical activity. Moreover, maternal role modelling was positively associated with physical activity in three studies [44, 58, 61]. A range of modifiable family- and childcare-related elements also showed positive associations with change in young children’s activity in fewer studies. Where positive effects on change in physical activity were seen, they were often small in magnitude, particularly in studies reporting accelerometer-measured outcomes. Despite identifying a range of determinants that have been assessed, there appears to be little evidence of what elements effect positive change in preschoolers’ physical activity. Where determinants have shown no positive effect (e.g. child/parental knowledge), researchers should divert emphasis instead to other potentially influential determinants. Both parental monitoring and maternal role modelling may provide feasible and effective determinants of change; given the lack of longitudinal evidence from the community and policy domains, and with no evidence to date from developing countries, further exploration of possible determinants of change in these areas is also required.

### Findings in the Context of Previous Research

As is also shown in cross-sectional studies [16, 25], the association between the child’s sex and change in physical activity [60, 61, 77–79] was not consistent here. In general, boys’ absolute levels of physical activity were reported to be higher than those of girls [61, 79], suggesting that, regardless of change, boys may remain more active than girls over time. The aim of this review was not to assess whether a determinant was associated with increased physical activity over time, but rather if different levels of a determinant predict differences in change in physical activity over time. Sex is a good example of this: boys’ physical activity may increase over time whilst girls’ activity remains stable, or boys’ activity may remain stable whilst girls’ activity decreases. Although the data available do not allow us to explore the actual direction of change, this is an important consideration for future research. Based on current evidence and quality of measurement, boys appear to be more active than girls, but firm conclusions about the influence of sex on changes in young children’s activity over time cannot be drawn.

Determinants in the interpersonal domain were most frequently assessed. Only one determinant, parental monitoring, was consistently positively associated with change in physical activity in both prospective and interventional studies in this age group. This was operationalized in a range of ways by increasing parental awareness of the child’s physical activity [66, 69], including using log books [44] and pedometers [42]. Although evidence of parental monitoring effecting a positive change in physical activity prospectively in older children is sparse [85, 86], cross-sectional evidence from a small sample of US children (*n* = 99) suggests that where parenting is permissive, parental monitoring may lead to increases in MVPA in children [87]. Evidence tends to suggest that parents tend to over-estimate their children’s physical activity in general [88]. Yet conscious parental monitoring of the target behaviour may increase its salience, resulting in a greater number of prompts to be active and therefore greater subsequent physical activity.

Three further studies reported a positive effect of maternal role modelling on children’s activity [44, 58, 61]; this ranged from assessing mothers’ own physical activity [61] to increasing maternal awareness and encouraging increased physical activity within families, with or without her child so as to model activity behaviour [44, 58]. These findings are supported by qualitative literature, with parents consistently suggesting that active parents and parents as role models were important facilitators of children’s activity [89–94]. Positive associations between parents’ and children’s activity have also been reported previously in cross-sectional studies [95–97]. Interventional studies targeting other interpersonal factors such as increasing parental knowledge [11, 42, 44, 48–50, 55, 56, 58, 64, 66, 69–73, 75, 76, 80–83] or social support [69, 72, 75, 83, 84], and improving parenting skills [49, 51, 76, 81] showed indeterminate associations; both high and lower quality studies reported both positive [42, 44, 49, 58, 66, 69, 83] and no associations [11, 48, 50, 51, 55, 56, 64, 70–73, 75, 76, 80–82, 84] for these interventional components. It may therefore be that it is parental awareness and their own activity behaviours that are important for their child’s activity. Further research is needed to explore how objectively measured physical activity in preschool-aged children and their parents are associated longitudinally.

Several reviews conducted previously suggest that elements in the preschool environment may be positively associated with children’s activity [27, 98]. Many interventional studies here specifically targeted the childcare environment, providing curriculum materials or modified elements within childcare settings, but no clear determinants were identified [11, 37, 39, 41, 43, 48–50, 53, 55, 56, 64, 71, 73, 75, 80]. Four of the interventional studies used variations of the same ‘Hip-Hop-to-Health’ intervention [11, 55, 56, 73], targeting a range of elements in the childcare setting: only one [56] showed a positive sustained effect on accelerometer-measured activity in a predominantly African American population. This highlights that even with a consistent core intervention, factors including cultural variability, differing reported outcomes and intervention fidelity likely influence intervention success.

Although environmental childcare determinants showed inconclusive results, of 16 interventional studies incorporating provider training, eight noted positive increases in children’s activity [38, 44, 46, 47, 49, 53, 54, 66] and MVPA in particular. Interestingly, those interventions showing positive effects often incorporated few additional environmental elements, including providing additional curriculum materials [49, 53]; they did however tend to include motor skill training [46, 47, 54, 66], or parental elements [44, 66], and/or allocate additional time for physical activity [38, 53, 66]. Introducing additional providers also led to increased physical activity in two out of three high-quality interventional studies, where external gym trainers [49] and professional coaches [65] led physical activity sessions.

Given the increasing amount of time children now spend in childcare, care providers feasibly play an important role in shaping children’s health behaviours. It is not possible here to disentangle which elements of training resulted in positive physical activity change, but encouraging care providers to build on their skill-base and/or confidence in multi-component interventions may be important. Moreover, qualitative literature suggests that care providers perceive themselves to be both a positive [99–101] and a negative [99, 102, 103] influence on children’s physical activity, yet no quantitative studies to date have specifically focused on care-providers own behaviour as a potential determinant. Doing so may be timely given providers believe they can influence children’s activity and that young children should be active, but many are not aware of how much physical activity young children require [104].

Despite an obvious lack of observational research informing intervention development, the majority of interventional studies (68%) were classified as multi-level [11, 42, 44, 46–48, 50–52, 54–56, 58, 63–76], targeting determinants across a range of domains. Though these studies used notionally similar exposures, e.g. targeting children, their parents and changing the preschool environment, inconsistent results were seen. As with all multi-faceted interventions, it is therefore difficult to tease out which components were effective and may explain in part why so few determinants were consistently associated with change in physical activity. Determinants across interpersonal and organisational levels may act synergistically or may counteract each other leading to null results. Although we attempted to determine how each interventional component influenced activity, no formal mediation analyses were identified and further exploration of how elements within an interventional result in positive change would be beneficial. For example, mixed-methods process evaluations may help to delineate determinants of children’s physical activity and aid future intervention development.

### Future Research Directions

This review highlights where research evidence and gaps exist. A large number of (interventional) studies have targeted determinants such as child motor/skills training; child and parental knowledge; provision of extra time for physical activity or curriculum materials; and provider training, with the studies overall showing no or indeterminate effects. Comparatively few studies have assessed a wide range of other determinants such as child/parent goal setting, and provider monitoring or social support. There is also a lack of studies assessing paternal determinants, and where this information is provided, studies tend to use maternal report. Only one determinant has been assessed in the community domain and none in the policy domain; no studies have been conducted to assess determinants in developing countries. Focusing research where such gaps exist will yield novel evidence, potentially prevent wastage of resources and promote physical activity change.

Moreover, little work has been conducted to explore how children’s activity levels change from infancy to the preschool period, with only six studies including children aged 2 years or younger [57, 58, 69, 70, 83, 84]. Questions remain about the optimal method for assessing physical activity in infants and toddlers [105]. Moreover, assessing physical activity across developmental periods may necessitate different measurement and processing protocols, complicating the assessment of change in physical activity. Nevertheless, given that the early years represent a period of rapid development and a crucial window for positive habit formation, it is important to determine for whom, how and why physical activity may change *throughout early childhood*, and whether behaviour and potential inequalities in health manifest and remain in later years.

Finally, determinants may be time or situation specific. Very few prospective observational studies have assessed determinants of physical activity change in young children. Including both prospective and interventional studies (and treating interventional components as determinants in the latter) allowed us to identify a wider range of factors that have been posited to effect change in physical activity. This review also indicates that determinants may differ within the same cohort depending on measurement method and follow-up period [i.e. in the CLAN study, there was no association between sex and counts per epoch at first follow-up [60] but a positive association between (male) sex and MVPA at second follow-up [61]]. Prospective studies allow assessment of change in behaviour over relatively long periods of time; interventions, with generally much shorter follow-up periods than prospective studies, may be able to capture more short-term fluctuations in behaviour. Both types of study also tend to assess differing types of determinants. Prospective studies have focused on child’s sex and parental psychosocial and temporal factors, whereas interventional studies target child skill and knowledge, parental knowledge and behaviour, and elements in the preschool environment including care-provider training and provision of curriculum materials. Both types of study are therefore beneficial to establish whether a determinant is associated with behaviour change, and whether change is sustained over time. In combination, a more comprehensive picture of the determinants’ landscape in children 0–6 years of age can emerge; this will ensure future research focuses on where gaps in the current evidence exist, whilst focusing work on areas where potential positive gains in changing young children’s physical activity are most likely to be made.

### Strengths and Limitations

This is the first systematic review, to our knowledge, to specifically explore determinants of change of physical activity in children aged 6 years and under across prospective cohort and interventional studies. Given that cohort and interventional studies offered the most appropriate design to extract determinants of change, our research strategy was restricted to prospective studies. We applied rigorous review methods and did not exclude papers based on language, but it is possible that all relevant publications may not have been included, as illustrated by the identification of an additional study at the data-extraction phase. As this review was restricted to published studies, publication bias cannot be discounted. One determinant (sex) was assessed in the same study twice and contributed by more than one paper [60, 61]; however, in general, our methods reduced potential bias by lending more weight to determinants assessed in four or more studies. The inclusion of a range of study types and measures of activity is both a strength and a limitation of this review; studies using pedometers and questionnaires tended to report positive interventional effects. Studies also used differing accelerometer cut points and adjusted for differing covariates in regression models. This heterogeneity highlights how differing study methods may influence findings and intervention success. All studies were conducted in high-income countries and approximately half of the studies had small final sample sizes (*n* < 50; studies = 15), which may have limited their statistical power to detect significant associations. Although we attempted to standardise outcomes across studies, five and 23 different outcome measures were used in prospective and interventional studies, respectively, preventing the use of meta-analysis here.

## Conclusions

This review identified a range of predominantly interpersonal and organisational determinants of change in young children’s physical activity; however, only parental monitoring of their child’s physical activity emerged as a consistently positive determinant of change, with provider training positively associated with change in children’s MVPA. Maternal role modelling was also positively associated with change in all three studies in which it was examined. Many determinants were explored in fewer than four studies, and multiple determinants were targeted within each interventional study. This heterogeneity in the determinants considered, and also in outcome measures used, limited the ability to identify consistent evidence for specific determinants. Future work should investigate potentially important lesser-explored or overlooked modifiable family- and childcare-related determinants; explore how determinants influence physical activity throughout the day and week; and deconstruct how the multiple elements within an intervention result in positive behaviour change. Assessment of determinants in the community and policy domains, in addition to studies conducted in developing countries, is also required. Such information will provide more robust evidence about the determinants of change in activity in preschool-aged young children, which is needed to inform the development of successful targeted interventions to increase activity levels in this population.

## Electronic supplementary material

Below is the link to the electronic supplementary material.

Supplementary material 1 (DOCX 132 kb)
