# Supplementary material for: Determinants of Change in Physical Activity in Children 0–6 years of Age: A Systematic Review of Quantitative Literature
Source: Sports Med. 2016 Dec 17;47(7):1349–74. doi: 10.1007/s40279-016-0656-0 (PMC5488114; doi:10.1007/s40279-016-0656-0)
Supplement: Supplementary file 1 — Supplementary material 1 (DOCX 132 kb) [file 40279_2016_656_MOESM1_ESM.docx]

Electronic Supplementary Material Table S1: Search strategy for full review and physical activity-specific update^a^

|  | | |
| --- | --- | --- |
| 1 | (Determin*4 or correlates or factors or predict*3 or associate*3 or interaction or influence*1 or temperament or beliefs or attitudes or knowledge or perceptions or views or intentions or facilitators or barriers or experiences or prevent*3 or reduc*5 or increas*3 or promot*3 or education or curriculum or program*3 or polic*3 or media or campaign or review or intervention*1 or initiative*1 or strategy*3 or evaluation or trial).mp. [mp=title, abstract, original title, name of substance word, subject heading word, protocol supplementary concept, rare disease supplementary concept, unique identifier] |  |
| 2 | (Infant* or Toddler* or Preschool* or Nurser*).mp. [mp=title, abstract, original title, name of substance word, subject heading word, protocol supplementary concept, rare disease supplementary concept, unique identifier] |  |
| 3^a^ | ((Fruit*1 or Vegetable*1 or juice or sugar sweetened beverage*1 or fizzy drinks or soft drinks or junk food or fast food or processed food or unhealthy food or takeaway food or non-core food or energy dense food or high fat food or fatty food or nutrient poor food or unhealthy diet or healthy eating or portion size or empty calories or confectionery or sweet*1 or dessert*1 or chocolate*1 or cake*1 or biscuit*1 or burger*1 or chip*1 or crisp*1 or snack*1 or breakfast or lunch or dinner or obes*6 or overweight).mp. [mp=title, abstract, original title, name of substance word, subject heading word, protocol supplementary concept, rare disease supplementary concept, unique identifier] |  |
| 4 | (physical activ*5 or inactiv*3 or exercise*1 or outdoor or TV or Television or Tele or sedentary or (screen adj time)).mp. [mp=title, abstract, original title, name of substance word, subject heading word, protocol supplementary concept, rare disease supplementary concept, unique identifier] |  |
| 5 | 1 AND 2 AND 3 AND 4 |  |
| 6 | 5 not (cerebral palsy or asthma or cystic fibrosis or autism).mp. [mp=title, abstract, original title, name of substance word, subject heading word, protocol supplementary concept, rare disease supplementary concept, unique identifier] |  |
| ^a^ | Search terms omitted in physical activity-specific update conducted in 2015 |  |

# Electronic Supplementary Material Table S2: Quality assessment criteria by study design

| Type of Study | Assessment Criteria | Operationalisation |
| --- | --- | --- |
| Prospective | - Sample recruitment - Measure of exposure - Measure of outcome - Number of participants - Participant retention - Analysis strategy | Representative of general population: 1  Objective measure used: 1 (subjective: 0)  Objective measure used: 1 (subjective: 0)  >100 participants: 1 (<50: low quality)  >70%: 1  Multivariable: 1 |
|  | Total possible score | 6 |
| Intervention | - Sample recruitment - Randomised design - Measure of exposure - Measure of outcome - Number of participants - Participant retention (>70%) - Analysis strategy | Representative of general population: 1  Randomisation of I/C groups: 1  Objective measure used: 1 (subjective: 0)  Objective measure used: 1 (subjective: 0)  >100 participants: 1 (<50: low quality)  >70%: 1  Multivariable: 1 |
|  | Total possible score | 7 |
